# Compensation Possibilities of Mental Disorders — Individual Case of a Child with Severe Neurological Disorder

**DOI:** 10.1192/j.eurpsy.2022.1108

**Published:** 2022-09-01

**Authors:** Y. Fedorova, N. Burlakova, Y. Mikadze

**Affiliations:** Lomonosov Moscow State University, Faculty of Psychology, Department Of Neuro- And Pathopsychology, Moscow, Russian Federation

**Keywords:** mental development, compensation, pediatric opsoclonus-myoclonus syndrome, developmental disorders

## Abstract

**Introduction:**

Neurological diseases often lead to mental disorders in children. After the structure of disorder is identified, an important task is to examine the abilities to compensate developmental delay (Lev S. Vygotsky).

**Objectives:**

The goal of the study was to explore potential of children with opsoclonus-myoclonus syndrome (OMS), which is important for psychocorrection.

**Methods:**

Case study of a boy with idiopathic OMS (aged 3 years 5 months) who was patient at the Psychoneurological Department no. 2 of the Russian Children Clinical Hospital. The following methods were used: analysis of anamnestic data, analysis of patient’s medical record, pathopsychological assessment.

**Results:**

Anamnestic data and medical records indicate the absence of pregnancy pathologies and normal early development. OMS was first detected when the child was aged 2 y. 7 m. Pathopsychological assessment gave the following results: 1) movement disorder (primary disorder) leads to secondary mental disorders; 2) locomotor activity disorders inhibit the child’s use of space and orientation of body in it; 3) secondary defects are detected in constructive activity, speech and drawing. Intact components of the mental processes: 1) the child demonstrates motivation for independent activity despite operational difficulties; 2) in certain activities, the general plan of actions remains intact, i.e., the goal set is actualized in movements and actions; 3) notions about objects, actions with them, planning and performance of movements are intact.

**Conclusions:**

The research demonstrates disbalance between operational difficulties and integrity of semantic orientation, internal planning of actions. The data prove the importance of discussion on abilities of children with OMS to compensate mental disorders.

**Disclosure:**

No significant relationships.

